# Detrimental Impact of Atrial Fibrillation among Patients Hospitalized for Acute Exacerbation of COPD: Results of a Population-Based Study in Spain from 2016 to 2021

**DOI:** 10.3390/jcm13102803

**Published:** 2024-05-09

**Authors:** Javier de-Miguel-Diez, Ana Lopez-de-Andres, José J. Zamorano-Leon, Valentín Hernández-Barrera, Natividad Cuadrado-Corrales, Ana Jimenez-Sierra, Rodrigo Jimenez-Garcia, David Carabantes-Alarcon

**Affiliations:** 1 Respiratory Care Department, Hospital General Universitario Gregorio Marañón, 28040 Madrid, Spain; javier.miguel@salud.madrid.org; 2 Department of Public Health & Maternal and Child Health, Faculty of Medicine, Universidad Complutense de Madrid, 28040 Madrid, Spain; josejzam@ucm.es (J.J.Z.-L.); mariancu@ucm.es (N.C.-C.); rodrijim@ucm.es (R.J.-G.); dcaraban@ucm.es (D.C.-A.); 3 Preventive Medicine and Public Health Teaching and Research Unit, Health Sciences Faculty, Rey Juan Carlos University, 28922 Madrid, Spain; valentin.hernandez@urjc.es; 4 Faculty of Medicine, Universidad San Pablo CEU, 28925 Madrid, Spain; a.jimenez100@usp.ceu.es

**Keywords:** atrial fibrillation, COPD, hospital admissions, prevalence, in-hospital mortality, sex differences

## Abstract

**Background/Objectives**: To analyze changes in the prevalence of atrial fibrillation (AF) in patients hospitalized for acute exacerbation of chronic obstructive pulmonary disease (AE-COPD); to evaluate hospital outcomes according to AF status, assessing sex differences; to identify factors associated with AF presence; and to analyze variables associated with in-hospital mortality (IHM) in AE-COPD patients with AF. **Methods**: We used data from the Registry of Specialized Care Activity-Basic Minimum Data Set (RAE-CMBD) to select patients aged ≥40 years with COPD in Spain (2016–2021). We stratified the study population according to AF presence and sex. The propensity score matching (PSM) methodology was employed to create comparable groups based on age, admission year, and comorbidities at the time of hospitalization. **Results**: We identified 399,196 hospitalizations that met the inclusion criteria. Among them, 20.58% had AF. The prevalence of AF rose from 2016 to 2021 (18.26% to 20.95%), though the increase was only significant in men. The median length of hospital stay (LOHS) and IHM were significantly higher in patients with AF than in those without AF. After PSM, IHM remained significantly higher for man and women with AF. Older age, male sex, and several comorbidities were factors associated with AF. Additionally, older age, male sex, different comorbidities including COVID-19, hospitalization in the year 2020, mechanical ventilation, and intensive care unit (ICU) admission were associated with higher IHM in patients with AE-COPD and AF. **Conclusions**: AF prevalence was high in patients hospitalized for AE-COPD, was higher in men than in women, and increased over time. AF presence was associated with worse outcomes. The variables associated with IHM in hospitalized AE-COPD patients with AF were older age, male sex, different comorbidities including COVID-19 presence, hospitalization in the year 2020, need of mechanical ventilation, and ICU admission.

## 1. Introduction

Chronic obstructive pulmonary disease (COPD) is a chronic respiratory disease characterized by irreversible obstruction and abnormal inflammation in the airways and can lead to the development of pulmonary heart disease and respiratory insufficiency [1]. It is one of the leading causes of mortality worldwide and generates important social and economic burdens for health systems [2].

Comorbidities are important, particularly in patients hospitalized for acute exacerbation of COPD (AE-COPD). Moreover, multimorbidity is a characteristic of these patients and influences the prognosis of COPD and AE-COPD [3].

Atrial fibrillation (AF) is a common comorbidity in patients with COPD [4], being the most common arrhythmia in them [5]. It is very often associated with other cardiac morbidities, particularly ischemic heart disease and heart failure. Moreover, COPD is associated independently with AF. In one large, retrospective cohort study carried out by Sidney et al. [6] that included 45,966 patients, they found a 4.41-times higher risk of AF in patients with COPD. In another study, the risk of comorbid AF increased in COPD patients, particularly during exacerbations [7]. However, the pathophysiological mechanisms that explain this association are complex and not completely clarified [8]. COPD and AF share common risk factors, which contribute to the beginning of both diseases [9]. Among the factors that contribute to AF development in COPD patients are tobacco consumption, oxidative stress, tissue hypoxia, and drug effects, including beta-adrenergic agonists, theophylline, and corticosteroids [10]. Both COPD and AF can potentially lead to a mutual exacerbation of the conditions, complicating their treatment in clinical practice and contributing significantly to the global health burden [11,12].

On the other hand, AF is associated with an accelerated decrease in lung function in COPD patients, as well as a significant increase in cardiovascular events, hospital admissions, and long-term mortality [6,13]. Severity of airflow obstruction is also related to the risk of new-onset AF [7]. So, AF prevalence is higher in patients with severe airflow obstruction in comparison to those with mild to moderate obstruction. Other risk factors for new-onset AF in COPD patients are related to demographic variables and cardiovascular diseases [14].

Despite the findings described, the majority of the investigations in which the relationship between COPD and AF have been evaluated have focused on patients with AF. By contrast, not many studies have been conducted on AF and hospitalized COPD [15]. However, the negative effects of AF on the outcomes of hospitalized AE-COPD are well known, and, in fact, the popular DECAF score for predicting exacerbations includes AF [15,16,17,18,19,20,21,22].

The objectives of our study were the following: to analyze changes in the prevalence of AF in AE-COPD patients in Spain during the period between 2016 and 2021; to describe and compare clinical characteristics and hospital outcomes according to AF status in AE-COPD patients, assessing sex differences; to identify factors independently associated with the presence of AF in patients hospitalized for AE-COPD; and to investigate the variables associated with in-hospital mortality (IHM) in AE-COPD patients with AF.

## 2. Materials and Methods

### 2.1. Research Approach, Target Group, and Data Analysis

A retrospective, observational, population-level study was undertaken, utilizing information obtained from hospital discharge summaries in the Spanish Registry of Specialized Care Activity-Basic Minimum Data Set (RAE-CMBD). This dataset compiles demographic and clinical details such as age, gender, dates of hospital admission, and discharge, along with up to 20 diagnoses and 20 medical procedures. The RAE-CMBD includes the data of patients hospitalized in all departments (medical and surgical), including intensive care units (ICU), for a minimum duration of 24 h. It excludes patients only treated in emergency departments without subsequent hospital admission. Additional information about the RAE-CMBD is available online [23].

The analysis covered the timeframe of 1 January 2016 to 31 December 2021. The hospital discharge reports were codified based on the International Classification of Diseases, Tenth Revision (ICD10).

The focus was on patients aged 40 and above with a primary diagnosis of AE-COPD. The primary diagnosis is defined as the main reason for hospitalization as determined by the attending physician at discharge. Patients with ICD10 code J41, J42, or J43 in the primary diagnostic position were considered to be admitted as a consequence of AE-COPD, as reported by Sadatsafavi et al. [24]. Excluded were COPD patients lacking complete data on gender, age, admission/discharge dates, or discharge destination.

The study grouped subjects by the occurrence of AF and sex. AF presence was identified if an ICD10 code for this condition (I48.0; I48.1; I48.2; I48.91) appeared in a secondary diagnostic field (2–20). We excluded 361 patients with an AF code and a “No” or “Unknown” indicator for present on admission (POA); this means that AF was first detected during the hospital admission.

The comorbidity assessment involved the Charlson Comorbidity Index (CCI) adapted for ICD10, as outlined by various authors [25,26].

Study variables also encompassed COVID-19 (for 2020 and 2021), asthma, pneumonia, bronchiectasis, influenza, tobacco use, obstructive sleep apnea, obesity, gastroesophageal reflux disease, hypertension, depression, anxiety, and thyroid disorders, as well as non-invasive and invasive ventilation. The corresponding ICD10 codes are shown in Table S1.

The hospitalization outcomes analyzed included ICU admissions, length of hospital stay (LOHS), and IHM.

### 2.2. Propensity Score Matching Methodology

Propensity score matching (PSM) was employed for creating comparable groups based on age, admission year, and comorbidities at the time of hospitalization. The PSM analyses involved comparisons between women and men, both with and without AF, using multivariable logistic regression as described by Austin [27]. Love plots to assess the effect of PSM can be seen in Figures S1 and S2.

### 2.3. Statistical Analysis

AF prevalence in AE-COPD patients was determined, segmented by year and sex.

Descriptive statistics were used, with categorical data presented as frequencies and percentages, and continuous data as either means with standard deviations (SD) or medians with interquartile ranges, based on distribution normality.

Time trends were examined using various statistical tests, including linear regression *t*-test, Cochran–Armitage, Cochran–Mantel–Haenszel, and Jonckheere–Terpstra, tailored to the data type.

Comparisons of categorical data utilized Fisher’s exact test, while continuous data comparisons used the *t*-test or Mann–Whitney test as appropriate.

Factors associated with the presence of AF in hospitalized AE-COPD patients, and the impact of AF on IHM, were analyzed using multivariable logistic regression, considering sex and other study variables. The recommendations of Hosmer et al. were applied for model construction [28].

### 2.4. Ethical Considerations

In Spain, access to the RAE-CMBD, managed by the Ministry of Health, requires researchers to submit a study proposal. If deemed ethically sound and clinically relevant, the Ministry provides the data [29]. Under Spanish law, patient consent is not required for this mandatory registry, ensuring patient anonymity.

## 3. Results

### 3.1. Hospitalization Trends and Atrial Fibrillation Prevalence in AE-COPD Patients

In Spain, from 2016 to 2021, there were 399,196 hospital admissions of patients aged ≥40 years diagnosed primarily with AE-COPD. Among these, 20.58% (82,164 cases) were also diagnosed with AF at the time of admission.

### 3.2. Temporal Patterns in AF Prevalence among AE-COPD Hospitalizations

**Figure 1:**
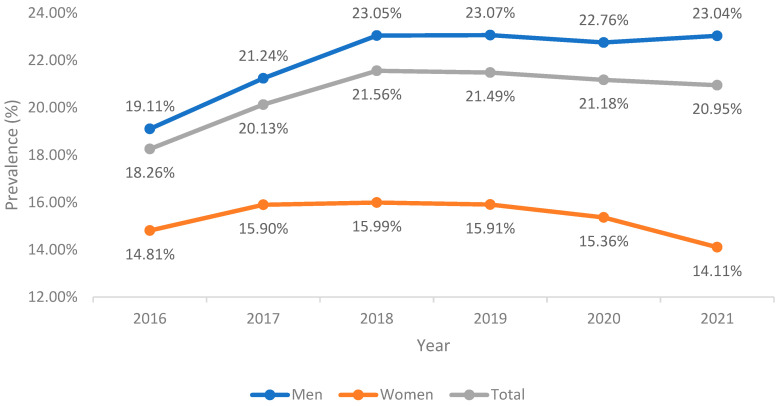
Time trend in the prevalence of atrial fibrillation among patients hospitalized with a primary diagnosis of acute exacerbation of COPD in Spain (2016–2021).

The overall prevalence of AF in hospitalized AE-COPD patients rose slightly from 2016 to 2021 (18.26% to 20.95%), as depicted in Figure 1. A consistent increase in AF prevalence was noted among men (from 19.11% to 23.04%; *p* < 0.001) but not among women (from 14.81% to 14.11%; *p* < 0.089). Figure 1 illustrates significantly higher AF prevalence in men across all study years.

### 3.3. Clinical Characteristics and Hospitalization Outcomes Based on AF Status in AE-COPD Patients

Table 1 shows that, over the entire time period, women with AF represented 15.98% of all patients with AF and 22.66% of patients without this condition.

Patients with AF had higher mean ages (79.58 vs. 73.17 years; *p* < 0.001) and a higher number of comorbidities according to the CCI score (1.47 vs. 0.96; *p* < 0.001). They had higher rates of COVID 19, pneumonia, obstructive sleep apnea, obesity, hyperthyroidism, and hypothyroidism, but lower rates of asthma, influenza, gastroesophageal reflux disease, anxiety, depression, hypertension, bronchiectasis, and smoking.

Both types of mechanical ventilation were less frequent in AF patients (0.9% and 4.8% vs. 1.14% and 4.98%; *p* < 0.001).

AF patients less often required intensive care unit admission (2.18% vs. 2.46%; *p* < 0.001) and had longer median hospital stays (7 days vs. 6 days; *p* < 0.001). Their crude in-hospital mortality (IHM) rate was higher (7.86% vs. 4.81%; *p* < 0.001).

### 3.4. Sex-Specific Hospitalization Characteristics and Outcomes in AE-COPD Patients with AF

As can be seen in Table 2, before PSM, women with AF hospitalized from 2016 to 2021 were, on average, 10 years older than those without AF. They had higher prevalence rates for several comorbidities, notably congestive heart failure, diabetes, and renal disease (all *p* < 0.001) and the mean CCI. The IHM was 7.26% for women with AF and 3.54% for those without (*p* < 0.001).

The use of PSM showed a good adjustment for the study variables (Figure S1) as the differences became not significant for most of them. However, after PSM, IHM remained significantly higher for women with AF (7.26% vs. 6.1%; *p* < 0.001).

Similar trends were observed among men, as shown in Table 3.

Men hospitalized from 2016 to 2021 with AF were on average five years older than those without AF (*p* < 0.001) and had higher comorbidity prevalence and mean CCI. The IHM was 7.98% for men with AF compared to 5.18% for those without (*p* < 0.001).

After an adequate adjustment with PSM (Figure S2), men with AF showed persistently higher IHM than those without AF (7.98% vs. 7.33%; *p* < 0.001).

### 3.5. Factors Linked to the Presence of AF in Hospitalized AE-COPD Patients

As detailed in Table 4, multivariable analysis identified older age, congestive heart failure, cerebrovascular disease, diabetes mellitus, renal disease, pneumonia, hypertension, obstructive sleep apnea, hypothyroidism, and hyperthyroidism as risk factors associated with AF at admission in both sexes.

Liver disease and obesity were linked to AF at admission only in men (OR 1.1, 95% CI 1.06–1.14 and OR 1.15, 95% CI 1.12–1.18). The time trend shown in the univariate analysis was confirmed in the multivariable, as a more recent year of hospital admission was associated with the presence of AF.

The presence of dementia, cancer and metastatic cancer, anxiety, and depression, were protective factors associated with AF in both sexes, while the presence of gastroesophageal reflux and bronchiectasis were protective factors associated with AF in men.

When both sexes were analyzed together, we found that men had a 35% higher likelihood of an AF diagnosis compared to women (OR 1.35; 95% CI 1.31–1.37).

### 3.6. Variables Associated with In-Hospital Mortality in AE-COPD Patients with AF

Table 5 reveals that older age, congestive heart failure, dementia, cancer, pneumonia, and COVID-19 presence were associated with higher IHM in both sexes in patients with AE-COPD and AF. Invasive and non-invasive mechanical ventilation and intensive care unit admissions also correlated with increased death risk. However, obstructive sleep apnea, hypothyroidism, and hyperthyroidism decreased IHM risk.

In men with AE-COPD and AF, cerebrovascular disease, liver disease, and renal disease significantly impacted IHM. However, the presence of diabetes, asthma, obesity, and hypertension decreased the risk of IHM.

In women with AE-COPD and AF, gastroesophageal reflux disease was associated with lower IHM.

Finally, hospitalization in the year 2020 was associated with a higher IHM in both men and women with AE-COPD and AF.

Woman with AF had lower IHM (OR 0.90; 95% CI 0.83–0.97) than men.

## 4. Discussion

Our study demonstrated a high prevalence of AF (20.58%) in patients hospitalized for AE-COPD, with a higher prevalence in male patients than in female. These results are in line with those reported by other authors, who have detected a high risk of developing AF in COPD patients [30], which is even higher in those with frequent exacerbations [31]. Using a nationwide cohort, Desai et al. [32] evidenced that 30% of patients hospitalized with COPD had arrhythmias, with AF being the most common subtype (22.1%). As in our case, male patients with COPD had arrhythmia more often than women with COPD. Abdullah et al. [15] also described a high prevalence of AF in this population of patients (16.6%), regardless of onset. Among the mechanisms that have been suggested to explain this association are the existence of common cardiovascular risk factors, such as tobacco consumption, underlying atherosclerosis, inflammation, heart failure, and obstructive sleep apnea. On the other hand, reduced pulmonary function, hypoxia, hypercapnia, and higher levels of pulmonary artery pressure, as well as the adverse effects of respiratory drugs, especially beta-agonists, have also been implicated in this relationship [15,33].

A high prevalence of AF has also been detected in hospitalized patients without COPD. Roten et al. [34] detected, in a large hospital-based population aged 65 to 84 years, a prevalence of clinical AF of 22.2%, which was also significantly higher in men than in women. In another study conducted in a large sample of hospitalizations for common cardiac and non-cardiac conditions, a high prevalence of AF was also detected, between 18.8 and 53.9% in White patients, with lower prevalence among Black and Hispanic patients across all admission categories [35]. In this study, the prevalence of AF in patients with COPD was similar to ours, specifically 20.4% in White patients.

Factors associated with the existence of AF in our study were older age, male sex, and some comorbidities, including congestive heart failure, cerebrovascular disease, diabetes mellitus, renal disease, pneumonia, obstructive sleep apnea, hypertension, hypothyroidism, and hyperthyroidism. Other investigators have also reported that patients hospitalized for EA-COPD with AF are older, less likely to be female, and have more comorbidities (especially heart failure, coronary artery disease, and obstructive sleep apnea) than those without AF [14,15].

Our analysis showed that prevalence of AF rose slightly from 2016 to 2021 in AE-COPD hospitalized patients. Xiao et al. [21] also found an increase in the AF prevalence in hospital encounters with end-stage COPD patients dependent on home oxygen therapy who were hospitalized with COPD exacerbation from 2003 to 2014. Factors that might be involved in this growing prevalence include aging population, increased AF awareness, advancing AF diagnostic approaches, and increasing trends in risk factors. By sex, a consistent increase in AF prevalence was noted among men in our study while not significant changes were observed in women.

The association between COPD and AF has marked implications in clinical practice. Indeed, it has been shown that the presence of AF exercises a detrimental impact on the outcomes of patients hospitalized for COPD exacerbation [10]. In our study, AF patients had a longer LOHS and higher HMI. The association between AF and mortality in EA-COPD patients is consistent with previous study findings, which have showed that the existence of AF behaves like an independent predictor for adverse outcomes, including mortality increase in COPD patients [15,36,37]. Nevertheless, the temporary sequence in the diagnosis is related to the prognosis, since if the diagnosis of COPD precedes that of AF, the risk of mortality is higher than when the diagnosis of COPD is determined after that of AF [38].

However, the temporary sequence in the diagnosis is related to the prognosis, since if the diagnosis of COPD precedes that of AF, the risk of mortality is higher than when the diagnosis of COPD is produced after that of AF.

Factors predictive of IHM in our study were older age, male sex, hospitalization in 2020, and different comorbidities, including COVID-19 infection, pneumonia, cancer, dementia, and congestive heart failure. In relation to the last factor, Tomioka et al. [39] have suggested that the existence of AF would cause additional deterioration of cardiopulmonary functions in patients with COPD, which would lead to high risk of congestive heart failure development with cardiac death. Other authors have also shown that coexisting cardiac comorbidities play a significant role in predicting arrhythmia and subsequent IHM in patients with COPD [32]. Likewise, need of mechanical ventilation and intensive care unit admissions were factors associated with increased death risk in our investigation, as has been previously described both in COPD patients [40] and in AF patients [41].

A highlighted finding in our study was the lower IHM in obese male patients (obesity paradox). The protective effect of obesity on IHM has been described previously in patients with COPD and AF [13]. Nevertheless, this phenomenon is considered to be controversial, as a high body mass index (BMI) is a marker of better nutritional status, less inflammation, and cachexia, while a low BMI is a marker of cachexia and/or frailty, promoting mortality [42].

There are several limitations in our study. It is a retrospective and observational study, based on registries and thus conducted using hospital discharge reports that include a very heterogenous group of clinical conditions [43,44], ranging from true respiratory exacerbations of COPD to exacerbations or respiratory symptoms caused by different diseases, pneumonia, pulmonary thromboembolism, and acute cardiovascular conditions including heart failure, ischemic heart disease, and AF. Furthermore, there is a lack of information in the study on the temporal evolution of atrial fibrillation—that is, whether it was present before or developed during hospitalized AE-COPD—as well as on the diagnosis and treatment of single episodes and during follow-up. As we use an administrative database, the diagnose of COPD was dependent on the ICD codes used. Thus, we cannot confirm said diagnosis or account for its severity with spirometry. Additionally, we did not have information on laboratory tests, image echocardiogram, or used medication, such as bronchodilators as a potential risk or use of beta-blockers or digitalis as protective treatments. However, our manuscript provides an exhaustive analysis of the relationship between COPD and AF in a large sample of patients hospitalized for AE-COPD and what may compensate for these limitations, strengthening the generalizability of our results.

## 5. Conclusions

In summary, we found that AF prevalence in patients hospitalized for AE-COPD was high, was higher in males than in females, and increased slightly from 2016 to 2021. The presence of AF was associated with a longer LOHS and a higher HMI. Factors independently associated with AF presence in these patients were older age, male sex, and several comorbidities. On the other hand, variables associated with IHM in AE-COPD patients with AF were older age, male sex, different comorbidities, hospitalization in the year 2020, need of mechanical ventilation, and intensive care unit admission. These findings indicate that the presence of AF is a detrimental event in patients admitted for AE-COPD. Thus, it is recommendable to establish a tailored management approach to improve the prognosis of the patients with both diseases.

## Figures and Tables

**Table 1:** Distribution of study covariates and hospital outcomes for people hospitalized with acute exacerbation of COPD in Spain from 2016 to 2021, according to the presence of atrial fibrillation.

	Atrial Fibrillation		
	Yes	No	*p*-Value
Women, n (%)	13,126 (15.98)	71,848 (22.66)	<0.001
Age, mean (SD)	79.58 (8.43)	73.17 (11.22)	<0.001
40–54 years old, n (%)	4248 (5.17)	73,547 (23.2)	<0.001
55–69 years old, n (%)	17,449 (21.24)	91,019 (28.71)	
70–84 years old, n (%)	34,608 (42.12)	98,534 (31.08)	
≥85 years old, n (%)	25,859 (31.47)	53,932 (17.01)	
CCI, mean (SD)	1.47 (1.13)	0.96 (1.03)	<0.001
Myocardial infarction, n (%)	5649 (6.88)	15,938 (5.03)	<0.001
Congestive heart failure, n (%)	37,070 (45.12)	54,371 (17.15)	<0.001
Peripheral vascular disease, n (%)	7591 (9.24)	22,592 (7.13)	<0.001
Cerebrovascular disease, n (%)	4436 (5.4)	12,401 (3.91)	<0.001
Dementia, n (%)	3520 (4.28)	11,150 (3.52)	<0.001
Diabetes mellitus, n (%)	27,635 (33.63)	83,860 (26.45)	<0.001
Rheumatoid disease, n (%)	1701 (2.07)	5958 (1.88)	<0.001
Mild/moderate/severe liver disease, n (%)	4907 (5.97)	22,919 (7.23)	<0.001
Renal disease, n (%)	20,567 (25.03)	41,737 (13.16)	<0.001
Cancer and metastatic cancer, n (%)	6605 (8.04)	26,129 (8.24)	<0.001
COVID-19, n (%)	949 (1.16)	3534 (1.11)	<0.001
Asthma, n (%)	3137 (3.82)	14,239 (4.49)	<0.001
Pneumonia, n (%)	14,117 (17.18)	42,964 (13.55)	<0.001
Influenza, n (%)	1250 (1.52)	5335 (1.68)	<0.001
Tobacco use, n (%)	42,012 (51.13)	204,613 (64.54)	<0.001
Obesity, n (%)	12,350 (15.03)	41,368 (13.05)	<0.001
Obstructive sleep apnea, n (%)	13,414 (16.33)	40,317 (12.72)	<0.001
Gastroesophageal reflux disease, n (%)	1132 (1.38)	5699 (1.8)	<0.001
Anxiety, n (%)	1935 (2.36)	12,843 (4.05)	<0.001
Depression, n (%)	2619 (3.19)	13,570 (4.28)	<0.001
Hypertension, n (%)	28,859 (35.12)	116,781 (36.84)	<0.001
Hypothyroidism, n (%)	4300 (5.23)	12,115 (3.82)	<0.001
Hyperthyroidism, n (%)	1633 (1.99)	3450 (1.09)	<0.001
Bronchiectasis, n (%)	3187 (3.88)	14,574 (4.6)	<0.001
Invasive lung ventilation, n (%)	738 (0.9)	3624 (1.14)	<0.001
Non-invasive lung ventilation, n (%)	3941 (4.8)	15,792 (4.98)	<0.001
Admission to intensive care unit, n (%)	1789 (2.18)	7806 (2.46)	<0.001
LOHS, median (IQR)	7 (6)	6 (5)	<0.001
IHM, n (%)	6461 (7.86)	15,240 (4.81)	<0.001

SD: Standard deviation; CCI: Charlson comorbidity index; LOHS: Length of hospital stay; IQR: Inter quartile range; IHM: In-hospital mortality.

**Table 2:** Distribution of study covariates and hospital outcomes for women with acute exacerbation of COPD in Spain from 2016 to 2021, according to the presence of atrial fibrillation before and after propensity score matching (PSM).

	Atrial Fibrillation before PSM			Atrial Fibrillation after PSM		
	Yes	No	*p*-Value	Yes	No	*p*-Value
Age, mean (SD)	80.13 (9.22)	69.91 (12.15)	<0.001	80.13 (9.22)	80.16 (10.02)	0.783
40–54 years old, n (%)	905 (6.89)	26,490 (36.87)	<0.001	905 (6.89)	908 (6.92)	0.156
55–69 years old, n (%)	2506 (19.09)	20,110 (27.99)		2506 (19.09)	2478 (18.88)	
70–84 years old, n (%)	4800 (36.57)	14,607 (20.33)		4800 (36.57)	4496 (34.25)	
≥85 years old, n (%)	4915 (37.44)	10,641 (14.81)		4915 (37.44)	5244 (39.95)	
CCI, mean (SD)	1.34 (1.05)	0.72 (0.91)	<0.001	1.34 (1.05)	1.32 (1.05)	0.058
Myocardial infarction, n (%)	520 (3.96)	1927 (2.68)	<0.001	520 (3.96)	524 (3.99)	0.899
Congestive heart failure, n (%)	6640 (50.59)	11,201 (15.59)	<0.001	6640 (50.59)	6558 (49.96)	0.311
Peripheral vascular disease, n (%)	475 (3.62)	2046 (2.85)	<0.001	475 (3.62)	466 (3.55)	0.765
Cerebrovascular disease, n (%)	643 (4.9)	1893 (2.63)	<0.001	643 (4.9)	556 (4.24)	0.010
Dementia, n (%)	743 (5.66)	2438 (3.39)	<0.001	743 (5.66)	750 (5.71)	0.852
Diabetes mellitus, n (%)	4087 (31.14)	15,168 (21.11)	<0.001	4087 (31.14)	4135 (31.5)	0.523
Rheumatoid disease, n (%)	392 (2.99)	2007 (2.79)	0.220	392 (2.99)	374 (2.85)	0.509
Mild/moderate/severe liver disease, n (%)	555 (4.23)	4401 (6.13)	<0.001	555 (4.23)	478 (3.64)	0.015
Renal disease, n (%)	3007 (22.91)	6362 (8.85)	<0.001	3007 (22.91)	2928 (22.31)	0.244
Cancer and metastatic cancer, n (%)	447 (3.41)	3225 (4.49)	<0.001	447 (3.41)	411 (3.13)	0.211
COVID-19, n (%)	115 (0.88)	671 (0.93)	0.525	115 (0.88)	171 (1.3)	0.001
Asthma, n (%)	1561 (11.89)	7934 (11.04)	0.004	1561 (11.89)	1508 (11.49)	0.309
Pneumonia, n (%)	1897 (14.45)	7706 (10.73)	<0.001	1897 (14.45)	1781 (13.57)	0.039
Influenza, n (%)	226 (1.72)	1385 (1.93)	0.112	226 (1.72)	203 (1.55)	0.263
Tobacco use, n (%)	4051 (30.86)	43,986 (61.22)	<0.001	4051 (30.86)	4038 (30.76)	0.862
Obesity, n (%)	2749 (20.94)	12,877 (17.92)	<0.001	2749 (20.94)	2803 (21.35)	0.414
Obstructive sleep apnea, n (%)	1604 (12.22)	7571 (10.54)	<0.001	1604 (12.22)	1594 (12.14)	0.850
Gastroesophageal reflux disease, n (%)	259 (1.97)	1687 (2.35)	0.008	259 (1.97)	251 (1.91)	0.721
Anxiety, n (%)	696 (5.3)	6482 (9.02)	<0.001	696 (5.3)	691 (5.26)	0.890
Depression, n (%)	987 (7.52)	6320 (8.8)	<0.001	987 (7.52)	955 (7.28)	0.450
Hypertension, n (%)	4639 (35.34)	26,225 (36.5)	0.011	4639 (35.34)	4872 (37.12)	0.003
Hypothyroidism, n (%)	1482 (11.29)	5905 (8.22)	<0.001	1482 (11.29)	1359 (10.35)	0.015
Hyperthyroidism, n (%)	399 (3.04)	1206 (1.68)	<0.001	399 (3.04)	343 (2.61)	0.037
Bronchiectasis, n (%)	452 (3.44)	2720 (3.79)	0.057	452 (3.44)	439 (3.34)	0.658
Invasive lung ventilation, n (%)	126 (0.96)	1026 (1.43)	<0.001	126 (0.96)	126 (0.96)	1.000
Non-invasive lung ventilation, n (%)	672 (5.12)	4473 (6.23)	<0.001	672 (5.12)	695 (5.29)	0.523
Admission to intensive care unit, n (%)	293 (2.23)	2199 (3.06)	<0.001	293 (2.23)	273 (2.08)	0.395
LOHS, median (IQR)	7 (6)	6 (5)	<0.001	7 (6)	7 (6)	0.083
IHM, n (%)	953 (7.26)	2544 (3.54)	<0.001	953 (7.26)	801 (6.1)	<0.001

SD: Standard deviation; CCI: Charlson comorbidity index; LOHS: Length of hospital stay; IQR: Inter quartile range; IHM: In-hospital mortality.

**Table 3:** Distribution of study covariates and hospital outcomes for men with acute exacerbation of COPD in Spain from 2016 to 2021, according to the presence of atrial fibrillation before and after propensity score matching (PSM).

	Atrial Fibrillation before PSM			Atrial Fibrillation after PSM		
	Yes	No	*p*-Value	Yes	No	*p*-Value
Age, mean (SD)	79.48 (8.27)	74.12 (10.75)	<0.001	79.48 (8.27)	79.36 (8.71)	0.328
40–54 years old, n (%)	3343 (4.84)	47,057 (19.19)	<0.001	3343 (4.84)	3409 (4.94)	0.265
55–69 years old, n (%)	14,943 (21.64)	70,909 (28.92)		14,943 (21.64)	14,588 (21.13)	
70–84 years old, n (%)	29,808 (43.18)	83,927 (34.23)		29,808 (43.18)	29,375 (42.55)	
≥85 years old, n (%)	20,944 (30.34)	43,291 (17.66)		20,944 (30.34)	21,666 (31.38)	
CCI, mean (SD)	1.49 (1.14)	1.02 (1.05)	<0.001	1.49 (1.14)	1.48 (1.15)	0.313
Myocardial infarction, n (%)	5129 (7.43)	14,011 (5.71)	<0.001	5129 (7.43)	5211 (7.55)	0.402
Congestive heart failure, n (%)	30,430 (44.08)	43,170 (17.61)	<0.001	30,430 (44.08)	30,157 (43.68)	0.139
Peripheral vascular disease, n (%)	7116 (10.31)	20,546 (8.38)	<0.001	7116 (10.31)	7052 (10.21)	0.570
Cerebrovascular disease, n (%)	3793 (5.49)	10,508 (4.29)	<0.001	3793 (5.49)	3658 (5.3)	0.108
Dementia, n (%)	2777 (4.02)	8712 (3.55)	<0.001	2777 (4.02)	2747 (3.98)	0.680
Diabetes mellitus, n (%)	23,548 (34.11)	68,692 (28.02)	<0.001	23,548 (34.11)	23,869 (34.57)	0.069
Rheumatoid disease, n (%)	1309 (1.9)	3951 (1.61)	<0.001	1309 (1.9)	1216 (1.76)	0.062
Mild/moderate/severe liver disease, n (%)	4352 (6.3)	18,518 (7.55)	<0.001	4352 (6.3)	3970 (5.75)	<0.001
Renal disease, n (%)	17,560 (25.44)	35,375 (14.43)	<0.001	17,560 (25.44)	17,457 (25.29)	0.524
Cancer and metastatic cancer, n (%)	6158 (8.92)	22,904 (9.34)	0.001	6158 (8.92)	6161 (8.92)	0.977
COVID-19, n (%)	834 (1.21)	2863 (1.17)	0.385	834 (1.21)	1098 (1.59)	<0.001
Asthma, n (%)	1576 (2.28)	6305 (2.57)	<0.001	1576 (2.28)	1396 (2.02)	0.001
Pneumonia, n (%)	12,220 (17.7)	35,258 (14.38)	<0.001	12,220 (17.7)	11,973 (17.34)	0.080
Influenza, n (%)	1024 (1.48)	3950 (1.61)	0.017	1024 (1.48)	940 (1.36)	0.056
Tobacco use, n (%)	37,961 (54.99)	160,627 (65.51)	<0.001	37,961 (54.99)	38,271 (55.43)	0.093
Obesity, n (%)	9601 (13.91)	28,491 (11.62)	<0.001	9601 (13.91)	8923 (12.92)	<0.001
Obstructive sleep apnea, n (%)	11,810 (17.11)	32,746 (13.36)	<0.001	11,810 (17.11)	11,752 (17.02)	0.678
Gastroesophageal reflux disease, n (%)	873 (1.26)	4012 (1.64)	<0.001	873 (1.26)	785 (1.14)	0.030
Anxiety, n (%)	1239 (1.79)	6361 (2.59)	<0.001	1239 (1.79)	1112 (1.61)	0.008
Depression, n (%)	1632 (2.36)	7250 (2.96)	<0.001	1632 (2.36)	1555 (2.25)	0.168
Hypertension, n (%)	24,220 (35.08)	90,556 (36.93)	<0.001	24,220 (35.08)	25,042 (36.27)	<0.001
Hypothyroidism, n (%)	2818 (4.08)	6210 (2.53)	<0.001	2818 (4.08)	2543 (3.68)	<0.001
Hyperthyroidism, n (%)	1234 (1.79)	2244 (0.92)	<0.001	1234 (1.79)	1068 (1.55)	<0.001
Bronchiectasis, n (%)	2735 (3.96)	11,854 (4.83)	<0.001	2735 (3.96)	2767 (4.01)	0.660
Invasive lung ventilation, n (%)	612 (0.89)	2598 (1.06)	<0.001	612 (0.89)	602 (0.87)	0.773
Non-invasive lung ventilation, n (%)	3269 (4.74)	11,319 (4.62)	0.191	3269 (4.74)	3014 (4.37)	0.001
Admission to intensive care unit, n (%)	1496 (2.17)	5607 (2.29)	0.061	1496 (2.17)	1446 (2.09)	0.351
LOHS, median (IQR)	7 (6)	6 (5)	<0.001	7 (5)	6 (5)	0.061
IHM, n (%)	5508 (7.98)	12,696 (5.18)	<0.001	5508 (7.98)	5059 (7.33)	<0.001

SD: Standard deviation; CCI: Charlson comorbidity index; LOHS: Length of hospital stay; IQR: Inter quartile range; IHM: In-hospital mortality.

**Table 4:** Multivariable analysis of factors associated with presenting atrial fibrillation diagnosed among patients hospitalized with acute exacerbation of COPD in Spain from 2016 to 2021, according to sex.

	Men	Women	Both
	OR (95% CI)	OR (95% CI)	OR (95% CI)
40–54 years old	1	1	1
55–69 years old	2.63 (2.53–2.74)	2.99 (2.76–3.24)	2.76 (2.67–2.87)
70–84 years old	4.11 (3.95–4.27)	5.72 (5.29–6.2)	4.48 (4.33–4.64)
≥85 years old	5.04 (4.83–5.25)	6.19 (5.69–6.74)	5.44 (5.24–5.64)
Congestive heart failure	2.99 (2.93–3.05)	3.34 (3.19–3.49)	3.07 (3.01–3.12)
Cerebrovascular disease	1.08 (1.04–1.12)	1.35 (1.22–1.49)	1.11 (1.07–1.15)
Dementia	0.86 (0.82–0.9)	0.8 (0.73–0.88)	0.85 (0.82–0.89)
Diabetes mellitus	1.06 (1.04–1.08)	1.16 (1.11–1.22)	1.08 (1.06–1.1)
Mild/moderate/severe liver disease	1.1 (1.06–1.14)	1.04 (0.94–1.15)	1.1 (1.06–1.14)
Renal disease	1.29 (1.26–1.32)	1.31 (1.24–1.39)	1.29 (1.26–1.32)
Cancer and metastatic cancer	0.96 (0.93–0.99)	0.88 (0.79–0.99)	0.95 (0.92–0.98)
Pneumonia	1.16 (1.13–1.19)	1.21 (1.14–1.28)	1.17 (1.14–1.19)
Obesity	1.15 (1.12–1.18)	NS	1.13 (1.1–1.16)
Obstructive sleep apnea	1.31 (1.28–1.34)	1.13 (1.05–1.2)	1.29 (1.26–1.32)
Gastroesophageal reflux disease	0.78 (0.72–0.84)	NS	0.8 (0.75–0.86)
Anxiety	0.81 (0.76–0.86)	0.72 (0.66–0.79)	0.76 (0.73–0.8)
Depression	0.82 (0.78–0.87)	0.85 (0.79–0.92)	0.83 (0.79–0.87)
Hypertension	1.15 (1.13–1.18)	1.1 (1.05–1.15)	1.15 (1.13–1.17)
Hypothyroidism	1.4 (1.33–1.47)	1.3 (1.22–1.39)	1.35 (1.3–1.41)
Hyperthyroidism	1.82 (1.69–1.97)	1.57 (1.38–1.78)	1.76 (1.65–1.87)
Bronchiectasis	0.91 (0.87–0.95)	NS	0.93 (0.89–0.97)
2017	1.13 (1.09–1.16)	NS	1.12 (1.09–1.15)
2018	1.27 (1.23–1.31)	NS	1.26 (1.22–1.29)
2019	1.24 (1.2–1.27)	NS	1.23 (1.19–1.26)
2020	1.22 (1.18–1.26)	NS	1.21 (1.17–1.24)
2021	1.2 (1.16–1.24)	NS	1.17 (1.14–1.21)
Men	NA	NA	1.35 (1.32–1.37)

NA: not applicable; NS: Not significant.

**Table 5:** Multivariable analysis of factors associated with in-hospital mortality during admission for patients with acute exacerbation of COPD in Spain from 2016 to 2021, according to the presence of atrial fibrillation.

	Men	Women	Both
	OR (95% CI)	OR (95% CI)	OR (95% CI)
40–54 years old	1	1	1
55–69 years old	1.3 (1.07–1.58)	2.04 (1.28–3.25)	1.39 (1.16–1.66)
70–84 years old	2.01 (1.67–2.42)	2.51 (1.59–3.94)	2.09 (1.76–2.48)
≥85 years old	2.99 (2.47–3.61)	4.65 (2.94–7.35)	3.2 (2.69–3.8)
Congestive heart failure	1.25 (1.18–1.33)	1.16 (1–1.34)	1.24 (1.17–1.31)
Cerebrovascular disease	1.3 (1.17–1.45)	NS	1.28 (1.16–1.42)
Dementia	1.6 (1.42–1.79)	1.55 (1.23–1.97)	1.59 (1.43–1.77)
Diabetes mellitus	0.9 (0.84–0.95)	NS	NS
Rheumatoid disease	NS	NS	0.77 (0.63–0.94)
Mild/moderate/severe liver disease	1.17 (1.04–1.32)	NS	1.15 (1.03–1.28)
Renal disease	1.16 (1.09–1.24)	NS	1.15 (1.08–1.22)
Cancer and metastatic cancer	1.8 (1.65–1.96)	1.83 (1.34–2.5)	1.8 (1.66–1.95)
COVID-19	5.79 (4.89–6.84)	3.99 (2.54–6.27)	5.52 (4.72–6.46)
Asthma	0.73 (0.59–0.91)	NS	0.78 (0.67–0.91)
Pneumonia	1.46 (1.36–1.56)	1.72 (1.45–2.03)	1.49 (1.4–1.59)
Obesity	0.73 (0.66–0.8)	NS	0.75 (0.68–0.82)
Obstructive sleep apnea	0.69 (0.63–0.76)	0.76 (0.59–0.97)	0.7 (0.64–0.76)
Gastroesophageal reflux disease	NS	0.41 (0.2–0.85)	0.73 (0.57–0.95)
Hypertension	0.85 (0.79–0.91)	NS	0.86 (0.81–0.92)
Hypothyroidism	0.79 (0.68–0.93)	0.79 (0.63–1)	0.79 (0.7–0.9)
Hyperthyroidism	0.66 (0.51–0.85)	0.59 (0.36–0.96)	0.64 (0.52–0.81)
Invasive lung ventilation	3.94 (3.18–4.88)	6.31 (3.78–10.55)	4.19 (3.45–5.1)
Non-invasive lung ventilation	2.91 (2.62–3.23)	3 (2.36–3.82)	2.92 (2.65–3.21)
Admission to intensive care unit	3.05 (2.62–3.55)	1.81 (1.19–2.76)	2.85 (2.47–3.28)
2017	NS	NS	NS
2018	NS	NS	NS
2019	NS	NS	NS
2020	1.16 (1.04–1.29)	1.35 (1.05–1.75)	1.19 (1.07–1.31)
2021	NS	NS	NS
Women	NA	NA	0.9 (0.83–0.97)

NA: not applicable; NS: Not significant.

## Data Availability

Data is contained within the article or Supplementary Materials.

## Supplementary Materials

The following supporting information can be downloaded at https://www.mdpi.com/article/10.3390/jcm13102803/s1: Table S1: Codes of the International Classification of Diseases, Tenth Revision used for this investigation; Figure S1: Love plot showing the comparison of covariate values for women with acute exacerbation of COPD with and without atrial fibrillation: absolute standardized differences before and after propensity score matching (PSM); Figure S2: Love plot showing the comparison of covariate values for men with acute exacerbation of COPD with and without atrial fibrillation: absolute standardized differences before and after propensity score matching (PSM).
