# Supplementary material for: Detrimental Impact of Atrial Fibrillation among Patients Hospitalized for Acute Exacerbation of COPD: Results of a Population-Based Study in Spain from 2016 to 2021
Source: J Clin Med. 2024 May 9;13(10):2803. doi: 10.3390/jcm13102803 (PMC11121781; doi:10.3390/jcm13102803)

Table S1. Codes of the International Classification of Diseases 10th revisions used for this investigation

Diagnosis and procedures	ICD10 Codes
Acute exacerbation of COPD	J41, J42, J43, J44
Atrial fibrillation	I48.0; I48.1; I48.2; I48.91
Asthma	J45
COVID 19	B34.2, B97.29, U07.1
Pneumonia	J12 to J18
Influenza virus	J9 to J11
Tobacco use	Z72.0; Z87.89; F17.2xxx
Obesity	E66.x
Obstructive sleep apnea	G47.33
Gastroesophageal reflux disease	K21.0, K21.9
Anxiety	F41
Depression	F34.1, F43.21; F32.9
Hypertension	I10
Hyperthyroidism	E05
Hypothyroidism	Eo3.9
Bronchiectasis	J47
Invasive mechanical ventilation	5A1935Z 5A1945Z, 5A1955Z,
Non-invasive mechanical ventilation	5A09357, 5A09457, 5A09557

Figure S1. Love plot showing the comparison of covariate values for women with acute exacerbation of COPD with and without atrial fibrillation: absolute standardized differences before and after propensity score matching (PSM). Footnote: CCI Charlson comorbidity index.

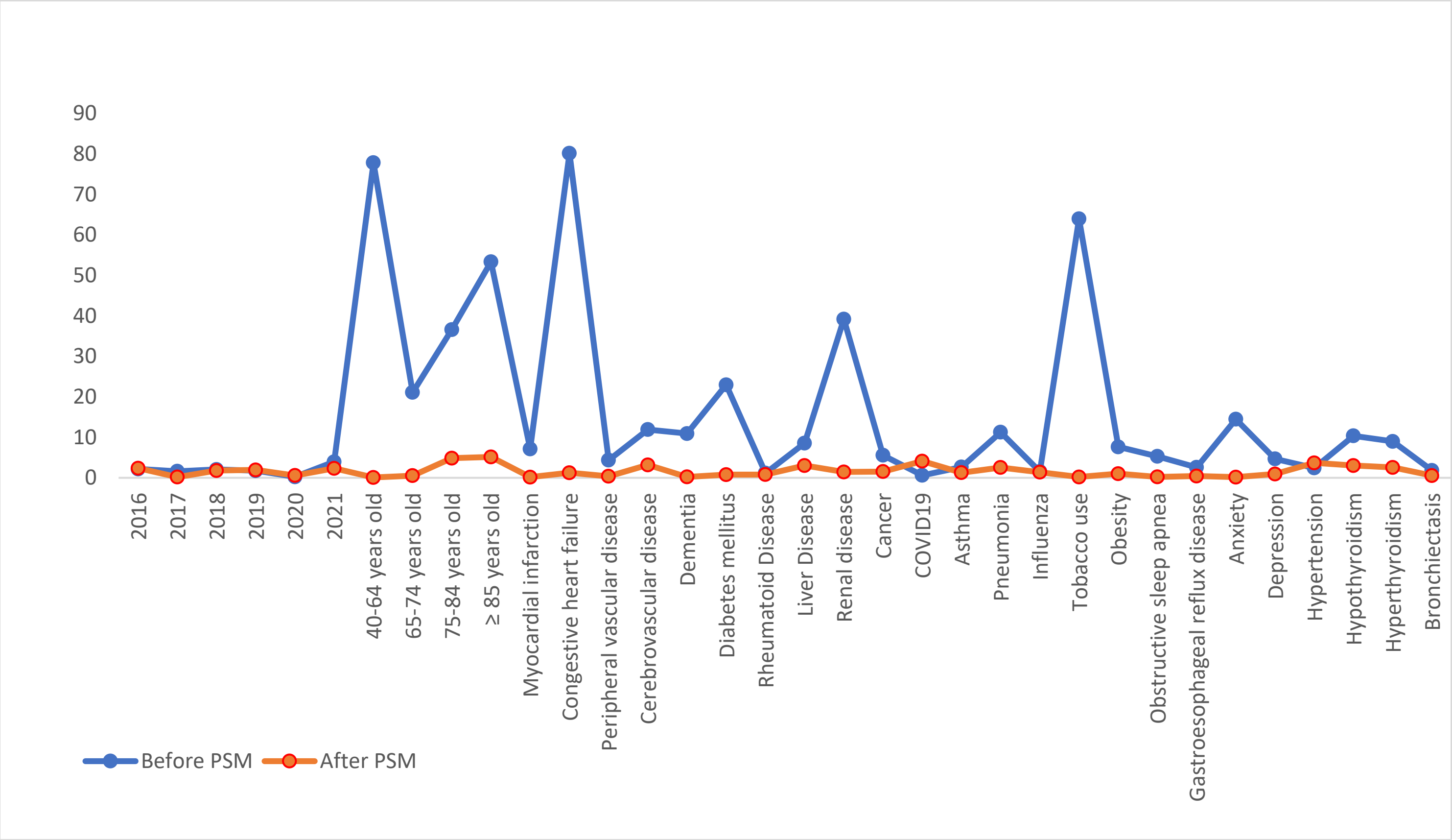

Supplementary Figure S2. Love plot showing the comparison of covariate values for men with acute exacerbation of COPD with and without atrial fibrillation: absolute standardized differences before and after propensity score matching (PSM). Footnote: CCI Charlson comorbidity index.

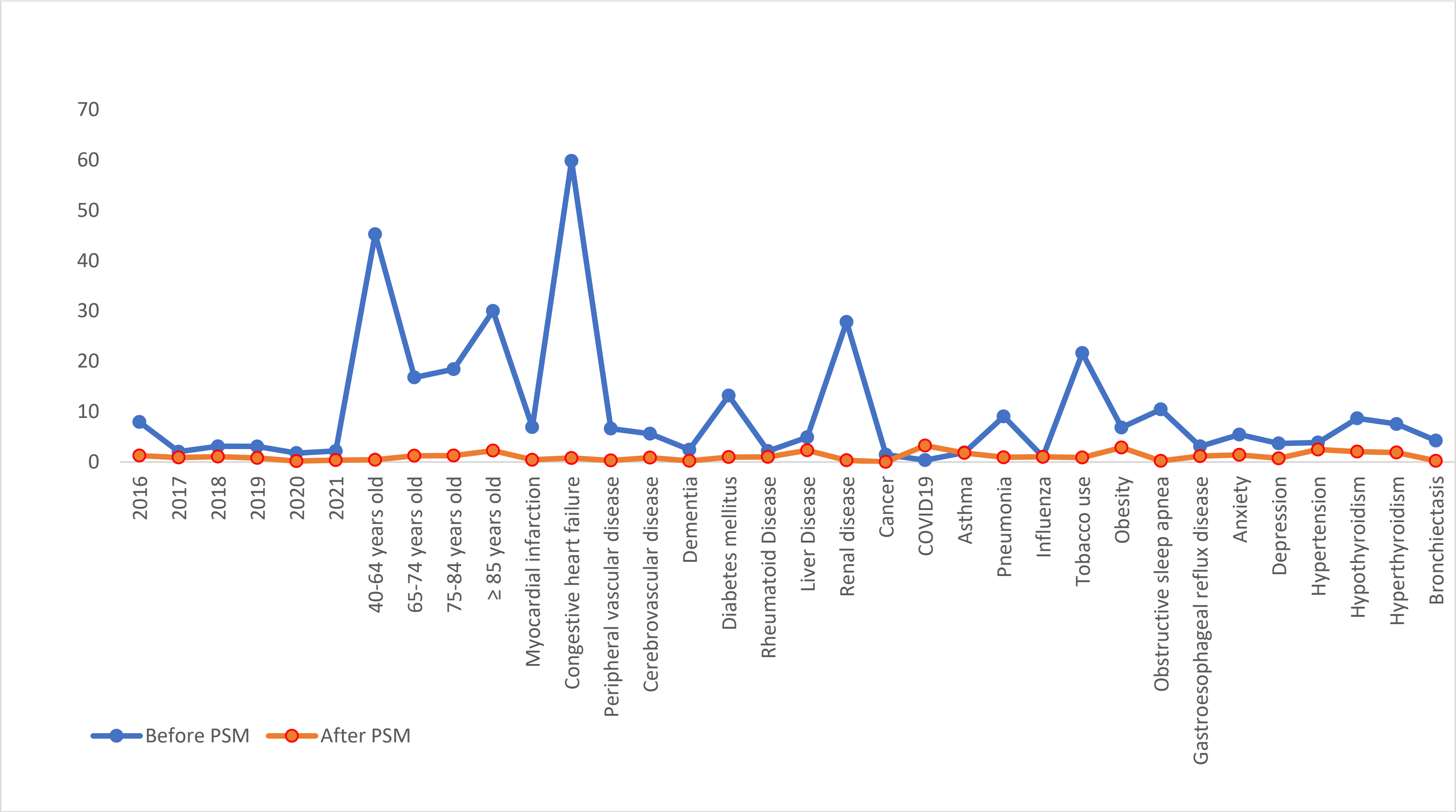

Supplement: Supplementary file 1 [file jcm-13-02803-s001.zip › jcm-3008551-supplementary.pdf]
